# Supplementary material for: Evaluation of deep learning for predicting rice traits using structural and single-nucleotide genomic variants
Source: Plant Methods. 2024 Aug 10;20:121. doi: 10.1186/s13007-024-01250-y (PMC11316328; doi:10.1186/s13007-024-01250-y)
Supplement: Supplementary file 1 — Additional file 1. [file 13007_2024_1250_MOESM1_ESM.docx]

**Supplemental Information**

**Evaluation of Deep Learning for predicting rice traits using structural and single-nucleotide genomic variants**

Ioanna-Theoni Vourlaki^1,4*^, Sebastián E. Ramos-Onsins^1^, Miguel Pérez-Enciso^1,2,3^_,_ Raúl Castanera^1,4*^

*^1^Centre for Research in Agricultural Genomics CSIC-IRTA-UAB-UB, Campus UAB, Edifici CRAG, Bellaterra, Barcelona 08193, Spain.*

*^2^Catalan Institute for Research and Advanced Studies (ICREA), Barcelona, Spain.*

*^3^Universitat Autónoma de Barcelona, Barcelona, 08193, Spain.*

*^4^* IRTA (Institut de Recerca i Tecnologia Agroalimentàries), 08140, Caldes de Montbui,

Barcelona, Spain

** For correspondence,* *ioanna.vourlaki@irta.cat,* [*raul.castanera@cragenomica.es*](mailto:raul.castanera@cragenomica.es)


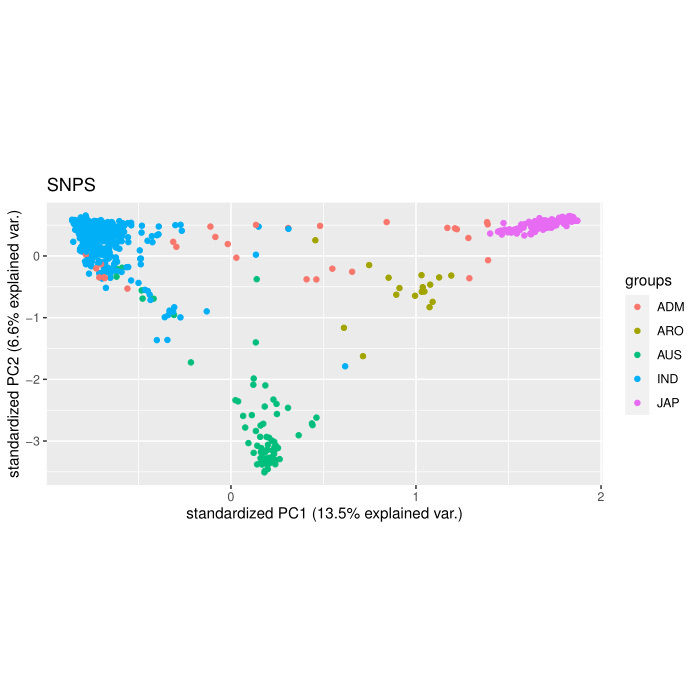


Figure 1: Principal components analysis applied to the SNP genotypic matrix. ADM = Admixture, ARO = Aromatic group, AUS = Aus group,IND = Indica group, JAP = Japonica group.


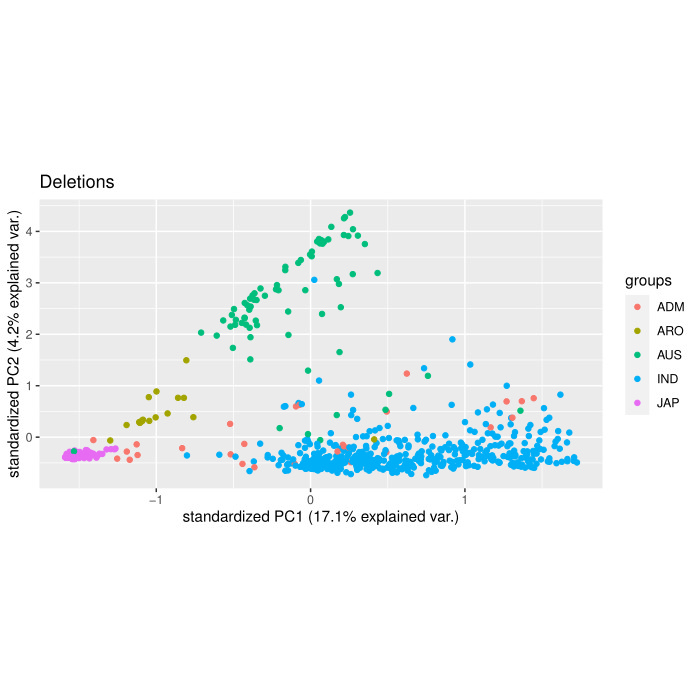


Figure 2: Principal components analysis applied to the DEL genotypic matrix. ADM = Admixture, ARO = Aromatic group, AUS = Aus group,IND = Indica group, JAP = Japonica group.


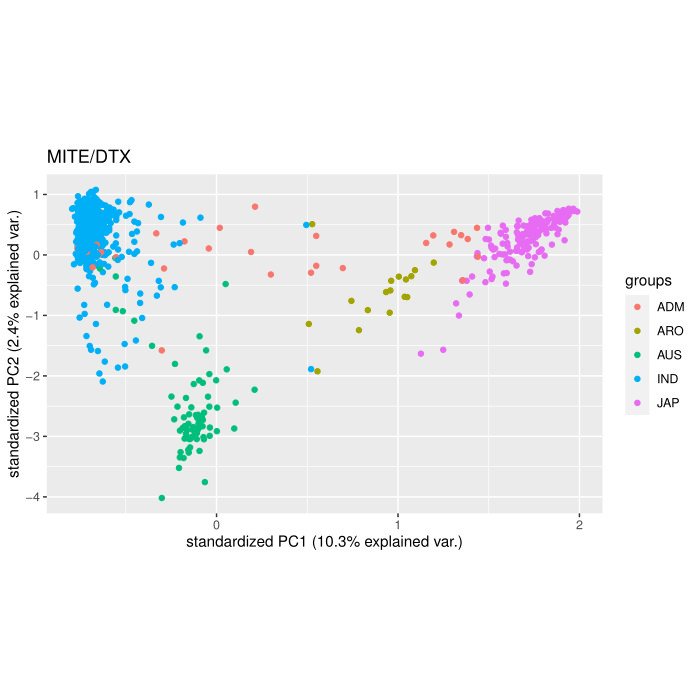


Figure 3: Principal components analysis applied to the MITE/DTX genotypic matrix. ADM = Admixture, ARO = Aromatic group, AUS = Aus group,IND = Indica group, JAP = Japonica group.


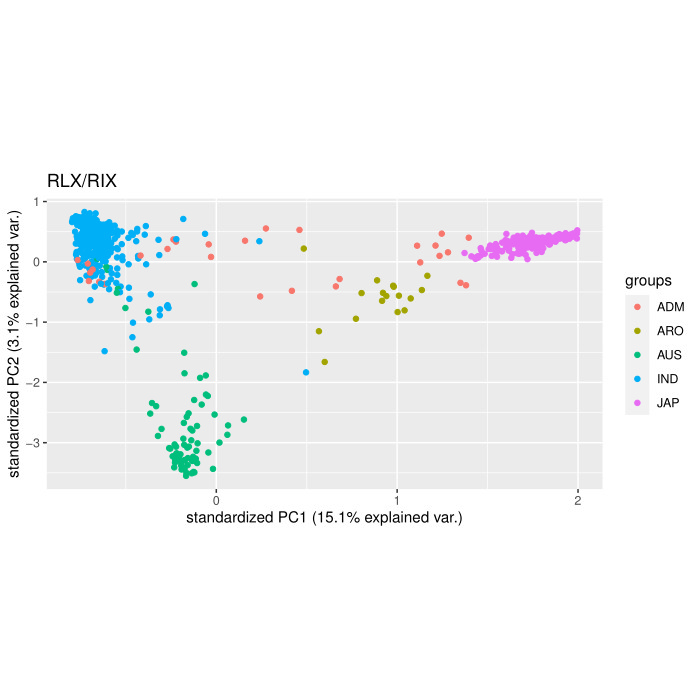


Figure 4: Principal components analysis applied to the RLX/RIX genotypic matrix. ADM = Admixture, ARO = Aromatic group, AUS = Aus group,IND = Indica group, JAP = Japonica group.


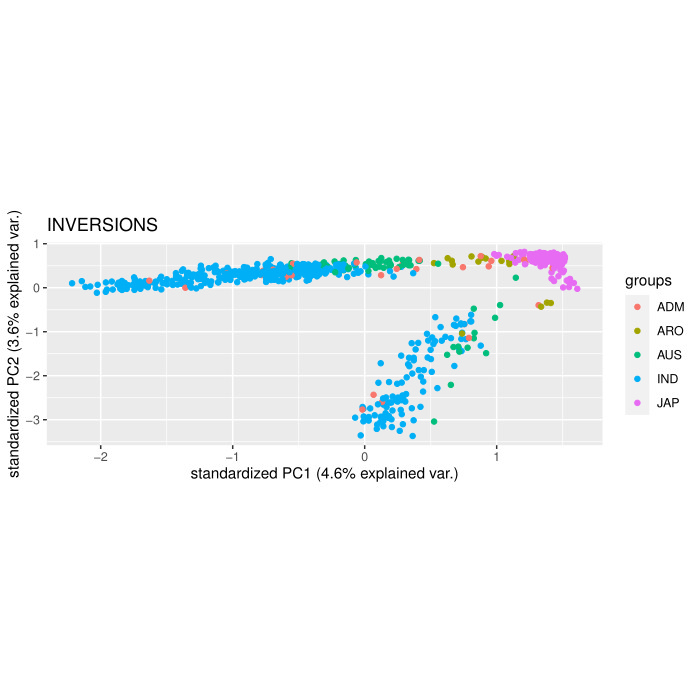


Figure 5: Principal components analysis applied to the INV genotypic matrix. ADM = Admixture, ARO = Aromatic group, AUS = Aus group,IND = Indica group, JAP = Japonica group.


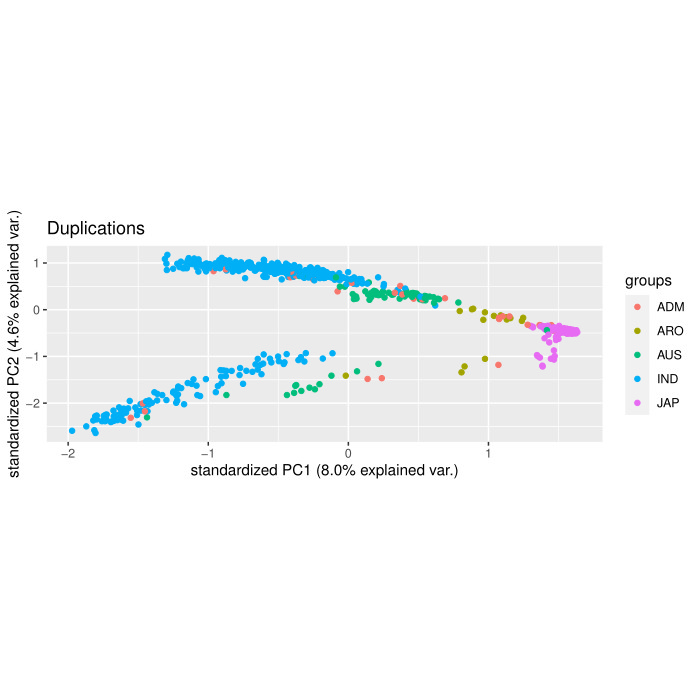


Figure 6: Principal components analysis applied to the DUP genotypic matrix. ADM = Admixture, ARO = Aromatic group, AUS = Aus group,IND = Indica group, JAP = Japonica group.

**Table 1:** Summary of the analyses performed.

|  | MLP, CNN, BayesC, RKHS, MLP PCs | | | RKHS, MLP PCs | |  |
| --- | --- | --- | --- | --- | --- | --- |
| Trait | **Combined variants** | **SNPs** | **Linked SNPs** | **Multiple Inputs** | |  |
| Culm diameter | x | x | x | x | x |  |
| Leaf senescence | x | x | x | x | x |  |
|  |  |  |  |  |  |  |
| Grain weight | x | x | x | x | x |  |
| Time to flowering | x | x | x | x | x |  |

**Best Hyperparameters for each trait**

The most frequently selected hyperparameters over four method categories, MLP, CNN, MLP with PCs and MLP with multiple inputs, for each of the studied traits are summarized in Tables 2-5. As tables depict, the optimal number of layers was 1 and 3 in 37.5% of the studied cases, followed by two layers with percentage of 25%. In the case of the activation function, hyperbolic tangent was the dominant in 50% of the summarized cases (8/16). Linear activation function was the second optimal choice with percentage around 37.5% whereas Rectified Linear Unit (Relu) selected in 12.5% of the cases. Among the available optimizers, Root Mean Square Propagation (RMSprop), Stochastic Gradient Descent (SGD) and Adaptive Moment Estimation (Adam), the most selected were Adam and RMSprop with percentage 43,75% each. In the case of dropout rate, the most frequent value to reduce overfitting in the model was 0.15 with 31.25 % followed by 0.05 with 24%. Concerning the number of filters, the two optimal values were 38 and 64 with equal frequency among the cases (50% each). Finally, we observed that for the number of neurons it was harder to point out one value since various numbers seem to be selected under different conditions. Note that in the case of MLP MULTIPLE, there are six hidden independent layers in which the input marker sets were forwarded. However, these do not count on the “No of hidden layers” (Tables 1-4) since this hyperparameter corresponds to the tuning after concatenating the six layers.

**Table 2:** Optimized hyperparameters for culm diameter.

| Hyperparameter | MLP | CNN | MLP PCs | MLP MULTIPLE |
| --- | --- | --- | --- | --- |
| Activation | Linear | Linear | Tanh | Tanh |
| No of hidden layers | 2 | 2 | 1 | 1 |
| No of neurons | (38,8) | (4,16) | (64) | (38) |
| No of filters | - | 64 | - | - |
| Optimizer | Adam | Adam | Adam | RMSprop |
| Dropout rate | 0.2 | 0.05 | 0.15 | 0.15 |
| Regularization | 0.001 | 0.01 | 0.01 | 0.001 |

**Table 3:** Optimized hyperparameters for leaf senescence.

| Hyperparameter | MLP | CNN | MLP PCs | MLP MULTIPLE |
| --- | --- | --- | --- | --- |
| Activation | Linear | Linear | Linear | Relu |
| No of hidden layers | 1 | 3 | 3 | 1 |
| No of neurons | (16) | (4,8,2) | (128,8,8) | (64) |
| No of filters | - | 38 | - | - |
| Optimizer | Adam | Adam | RMSprop | RMSprop |
| Dropout rate | 0.05 | 0.05 | 0.25 | 0.2 |
| Regularization | 0.001 | 0.001 | 0.01 | 0.001 |

**Table 4:** Optimized hyperparameters for grain weight.

| Hyperparameter | MLP | CNN | MLP PCs | MLP MULTIPLE |
| --- | --- | --- | --- | --- |
| Activation | Linear | Tanh | Tanh | Tanh |
| No of hidden layers | 1 | 3 | 2 | 3 |
| No of neurons | (38) | (16,16,16) | (128,16) | (64,8,2) |
| No of filters | - | 38 | - | - |
| Optimizer | Adam | Adam | RMSprop | RMSprop |
| Dropout rate | 0.1 | 0.05 | 0 | 0.25 |
| Regularization | 0.001 | 0.001 | 0.01 | 0.01 |

**Table 5:** Optimized hyperparameters for time to flowering.

| Hyperparameter | MLP | CNN | MLP PCs | MLP MULTIPLE |
| --- | --- | --- | --- | --- |
| Activation | Tanh | Tanh | Tanh | Relu |
| No of hidden layers | 1 | 3 | 3 | 2 |
| No of neurons | (16) | (2,8,16) | (128,2,8) | (128,8) |
| No of filters | - | 64 | - | - |
| Optimizer | SGD | SGD | RMSprop | RMSprop |
| Dropout rate | 0.1 | 0.05 | 0.1 | 0.25 |
| Regularization | 0.01 | 0.001 | 0.01 | 0.01 |
